# Effects of supervised exercise on cancer-related fatigue in breast cancer survivors: a systematic review and meta-analysis

**DOI:** 10.1186/s12885-015-1069-4

**Published:** 2015-02-21

**Authors:** José Francisco Meneses-Echávez, Emilio González-Jiménez, Robinson Ramírez-Vélez

**Affiliations:** 1 Grupo GICAEDS. Facultad de Cultura Física, Deporte y Recreación, Universidad Santo Tomás, Bogotá, D.C Colombia; 2 Departamento de Enfermería. Facultad de Ciencias de la Salud, Universidad de Granada, Granada, Spain

**Keywords:** Breast Neoplasms, Exercise, Resistance training, Rehabilitation, Medical oncology

## Abstract

**Background:**

Cancer-related fatigue (CRF) is the most common and distressing symptom in breast cancer survivors. Approximately 40% to 80% of cancer patients undergoing active treatment suffer from CRF. Exercise improves overall quality of life and CRF; however, the specific effects of the training modalities are not well understood.

**Methods:**

This study aimed to determine the pooled effects of supervised exercise interventions on CRF in breast cancer survivors. We searched PubMed/MEDLINE, EMBASE, Scopus, CENTRAL and CINAHL databases between December 2013 and January 2014 without language restrictions. Risk of bias and methodological quality were evaluated using the PEDro score. Pooled effects were calculated with a random-effects model according to the DerSimonian and Laird method. Heterogeneity was evaluated with the I^*2*^ test.

**Results:**

Nine high-quality studies (*n* = 1156) were finally included. Supervised aerobic exercise was statistically more effective than conventional care in improving CRF among breast cancer survivors (SMD = −0.51, 95%CI −0.81 to −0.21), with high statistical heterogeneity (*P* = 0.001; I^*2*^ = 75%). Similar effects were found for resistance training on CRF (SMD = −0.41, 95%CI −0.76 to −0.05; *P* = 0.02; I^2^ = 64%). Meta-regression analysis revealed that exercise volume parameters are closely related with the effect estimates on CRF. Egger’s test suggested moderate evidence of publication bias (*P* = 0.04).

**Conclusions:**

Supervised exercise reduces CRF and must be implemented in breast cancer rehabilitation settings. High-volume exercises are safe and effective in improving CRF and overall quality of life in women with breast cancer. Further research is encouraged.

**Trial Registration:**

CRD42014007223

## Background

Breast cancer is the most common cancer in women worldwide [1,2]. Breast cancer is also a leading cause of cancer death among women, accounting for 23% of total cancer cases and 14% of cancer deaths [3]. The World Health Organization (WHO) [4] estimated that breast cancer incidence in South America was 114,898 cases in 2008. In Colombia, nearly 5,000 new cases are diagnosed annually [5]. However, due to significant improvements in screening protocols, diagnosis, and treatment over the past few decades, breast cancer mortality has progressively decreased [6,7].

Cancer-related fatigue (CRF) is the most common and devastating symptom in breast cancer patients during and after therapeutic treatment [8]. Breast cancer patients continue to experience fatigue symptoms for months or years after successful treatment. Stone and colleagues observed that 75% of patients with various solid tumors (among whom 48 of 95 had metastatic disease) had a significantly increased cancer-related fatigue score compared with a matched control population [9]. It has been suggested that CRF might be considered a strong predictor of lower survival for cancer patients [10].

Exercise is widely recognized as an effective non-pharmacological therapy in cancer patients [11-13]. A growing body of evidence supports the idea that increasing physical activity provides important benefits to promote psychological outcomes and physical well-being in cancer patients [13-16]. These symptoms have been associated with clinical-related outcomes in breast cancer patients receiving active treatment regimens [11,13,17-24]. Some systematic reviews have communicated ambiguous findings concerning the effects of exercise interventions on CRF [11-13,15-22]. A recent Cochrane systematic review about exercise and CRF [17] concluded that aerobic exercise reduces CRF and encouraged further research of other exercise modalities. However, that review only included data published by March 2011 and did not examine supervised physical activity interventions in isolation from unsupervised interventions. A recent prospective randomized pilot trial by Oechsle et al. [25] reported that supervised exercise improved fatigue symptoms in 48 patients receiving myeloablative chemotherapy during the hospitalization period for chemotherapy. Nonetheless, the optimal doses and modes of exercise have not been addressed [26-28], yet these issues are essential to reach a more complete understanding of CRF control through supervised exercise training. In light of these gaps in the literature, this systematic review aimed to determine the pooled effects of supervised exercise interventions on CRF in breast cancer survivors, via a meta-analysis of randomized controlled trials.

## Methods

### Protocol and objective

This systematic review was conducted and reported in accordance with the Preferred Reporting Items for Systematic Reviews and Meta-Analyses (PRISMA) Statement [29] (PROSPERO Register code: CRD42014007223). No funding support was received in this study.

### Search methods

Two blinded authors (JFM-E and RR-V) independently applied the search strategy between December 2013 and January 2014. The electronic databases (PubMed/MEDLINE, EMBASE, Scopus, CENTRAL and CINAHL) databases were systematically searched by combining *Boolean operators* and any of the following search terms: “breast cancer”, “cancer-related fatigue” and “exercise”. (See Appendix 1 for further details). The authors incorporated the recommendations of Robinson and Dickersin [30] to achieve a highly sensitive search strategy for the retrieval of clinical trials on PubMed. The title and abstract were examined and full text was obtained if there was ambiguity regarding eligibility. In addition, the authors examined the reference lists of the identified records and the conference abstracts of the American Society of Clinical Oncology (ASCO) Annual Meeting on its website from 2004 to 2013, as well as certain journals (i.e., The Lancet Oncology, Journal of Clinical Oncology, Journal of the National Cancer Institute, Journal of Breast Cancer, The Breast Journal, and The Breast). No language restrictions were applied. Attempts were made to contact authors of trial reports if clarification was necessary.

### Ethics proclamations

This systematic review and meta-analysis included experimental studies that followed the provisions stated in the Declaration of Helsinki and were approved by the Ethics Committee. All patients signed informed consent. One author (JFM-E) performed this verification.

### Selection criteria

After screening the search results, two blinded authors (JFM-E and EGJ) independently evaluated eligibility of all studies retrieved from the databases based on the selection criteria. The studies were included if they met the following criteria according to the Patient/Problem, Intervention, Comparison/Control or Comparator and Outcomes/ Effects (PICO) methodology [29].

We included randomized controlled trials involving breast cancer survivors without restrictions to a particular stage of disease. Systematic reviews, editorials, cross-sectional studies, case reports and case series studies were excluded. We performed a subgroup analysis according to the stage of treatment for those studies involving participants during or after therapeutic anti-cancer treatment. Supervised exercise interventions were included in the systematic review, while non-supervised exercise programs were excluded. Exercise interventions were evaluated according to the definition of physical activity provided by Wolin et al. [31], “as any body movement causing an increase in energy expenditure that involves a planned or structured movement of the body performed in a systematic manner in terms of frequency, intensity, and duration and is designed to maintain or enhance health-related outcomes”. Therefore, tai-chi, manual therapy (joint mobilization techniques and therapeutic massage) and cognitive-behavioral interventions were excluded due to excessive variation in their mode, frequency, duration and intensity. Conventional care was considered a comparison group, and this group included women who did not participate in any exercise intervention program. Studies that compared supervised exercise with pharmacological and surgical treatments were excluded. Disagreements were resolved by consensus and the participation of a third author (RRV).

### Data extraction and quality assessment

Two authors (JFM-E and RRV) independently performed data extraction. Relevant data were extracted to a computer-based spreadsheet. The reviewers extracted the following information: authors’ information, publication year, study design, cancer treatment, time since diagnosis and characteristics of the exercise interventions (mode of training, length, duration and frequency) and effect estimates.

The methodological quality of the studies, including their risk of bias, was assessed using the PEDro scale, which is based on the Delphi list [32]. The PEDro scale scores the methodological quality of randomized trials out of 10. The score for each included study was determined by a trained assessor (JFM-E). Scores were based on all information available from both the published version and from communication with the authors. A score of 5 of 10 was set as the minimum score for inclusion in the current meta-analysis [33]. Three authors (JFM-E, RRV and EGJ) independently performed this assessment.

### Outcome measures

Cancer-related fatigue (CRF) was the primary outcome measure. The National Comprehensive Cancer Network (NCCN) [34] defines CRF as “a distressing, persistent, subjective sense of physical, emotional and/or cognitive tiredness or exhaustion related to cancer or cancer treatment that is not proportional to recent activity and interferes with usual functioning.” We considered the following validated tools for the measurement of fatigue levels: the Functional Assessment of Cancer Therapy (FACT)-Fatigue Scale, European Organization for Research and Treatment of Cancer Quality of Life Questionnaire (EORTC QLQ-C30), Piper Fatigue Scale (PFS), Schwartz Cancer Fatigue Scale (SCFS) and the Multidimensional Fatigue Inventory (MFI). Furthermore, we considered the following secondary outcome measures: depression; body mass index (BMI) as an indicator of body composition closely related to cancer progression; physical activity levels (minutes per week); and quality of life including physical, social, emotional and functional well-being. Pooled analysis for secondary outcomes was carried out if at least two studies were available for the outcome.

### Data synthesis

All statistical analyses were conducted using Comprehensive Meta-Analysis and Review Manager Software [35], developed by the Cochrane Collaboration. CRF was reported as continuous data. Therefore, we recorded both the mean change from baseline for each group or the mean post-intervention and standard deviation. Considering that different scales were used for the outcome measurements, we calculated standardized mean differences (SMD) with 95% confidence intervals (CI). If standard deviations were not reported, they were estimated through standard errors (CI or *t* values) [36]. SMDs were significant if their 95% CIs excluded zero. When high heterogeneity (I^2^ > 50%) was detected, the pooled effects were calculated by using a random-effects model reported in accordance with the DerSimonian and Laird method, which considers both within-study and between-study differences [36]. On the contrary, if substantial heterogeneity was not detected, we conducted a fixed-effects model reported by using the inverse variance method [36].

Statistical heterogeneity of the effect estimates among studies was assessed using I^2^ statistic which estimates the percentage of total variation across studies that was attributable to heterogeneity rather than to chance [37]; values greater than 50% were considered indicative of high heterogeneity. We performed a meta-regression analysis to explore the predictor effects of the supervised exercise characteristics, such as length (weeks), frequency (sessions per week), and duration (minutes per session) on the effect estimates. Publication bias was evaluated with the Egger’s test [36]. Two-sided *P* values of less than 0.05 were considered statistically significant.

## Results

### Characteristics of the studies included

**Figure 1:**
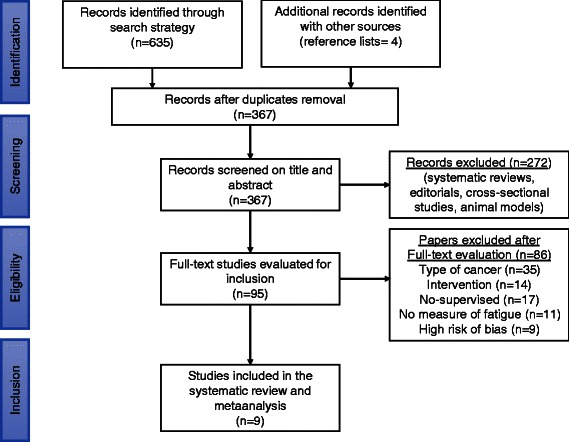
**Flowdiagram for search strategy methods.** Flowdiagram is performed according to PRISMA Statement.

Nine studies [38-46] (*n* = 1156) were included in the systematic review and meta-analysis. The assessment of risk of bias showed a mean PEDro score of 6.33 (SD = 1.1), indicating consistent methodological quality and a low risk of most biases (Table 1). The mean publication year for the included studies was 2008 (SD = 4.5), and most were conducted in North America (*k* = 3), United Kingdom, Finland, Australia and Turkey. Figure 1 presents the PRISMA flow diagram.

**Table 1:** **Assessment of methodological quality and risk of bias with PEDro scale**

Study	Random allocation	Concealed allocation	Groups similar at baseline	Participant blinding	Therapist blinding	Assessor blinding	<15% dropouts	Intention to treat analysis	Between-group difference reported	Point estimate and variability reported	Total (0 to 10)
Campbell et al. 2005 [38]	Y	N	Y	N	N	N	Y	N	Y	Y	5
Cantarero et al. 2013 [39]	Y	Y	Y	N	N	Y	Y	N	Y	Y	7
Courneya et al. 2003 [40]	Y	N	Y	N	N	Y	Y	Y	Y	Y	7
Ergun et al. 2013 [41]	Y	N	Y	N	N	Y	Y	N	Y	Y	6
Milne et al. 2008 [42]	Y	Y	Y	N	N	N	Y	Y	Y	Y	7
Mutrie et al. 2007 [43]	Y	Y	Y	N	N	Y	Y	Y	Y	Y	8
Saarto et al. 2012 [44]	Y	N	Y	N	N	N	Y	N	Y	Y	5
Segal et al. 2001 [45]	Y	N	Y	N	N	N	N	Y	Y	Y	5
Winters et al. 2012 [46]	Y	Y	Y	N	N	Y	N	Y	Y	Y	7

### Characteristics of breast cancer survivors

The mean age of participants in the included studies ranged from 48 to 60 years with an average of 55.2 years old (SD = 4.1). Breast cancer survivors were predominately non-Hispanic whites. Supervised exercise training groups comprised a total of 556 breast cancer survivors, and 460 women were allocated to control groups. Sixty-seven percent (*n* = 6 studies) [38,40,42-45] of the studies were conducted during active treatment, including chemotherapy and radiotherapy regimens. Regarding treatment descriptions, 638 participants received chemotherapy, and 510 received radiotherapy. The studies rarely reported time since diagnosis. Table 2 summarizes the characteristics of the studies included.

**Table 2:** **Characteristics of the studies included**

Study	Design	Breast cancer description	Participants*	Intervention**	Outcome measures
Campbell et al. 2005 [38]	RCT	Early stage (I-II) Breast cancer	Characteristics of cancer treatment = Chemotherapy, radiotherapy and combination.	Exp = Aerobic exercise and resistance training	FACT-G, FACT-B, SWLS, PFS, SPAQ, 12-minute walk test
			N = 22	Length = 12 weeks.	
			Female = 22	Duration = 20 min/session	
			Exp (n = 12)	Frequency = 2 session/week	
			Age (yr) = 48 (10)	Intensity = 60%-75%	
			Age (yr) = 47 (5)	Con = Conventional care	
Cantarero et al. 2013 [39]	RCT	Breast cancer (stages I-IIIA)	Characteristics of cancer treatment = Chemotherapy radiotherapy and combination.	Exp = Aerobic exercise and resistance training	PFS, The Spanish version of the Profile of
				Length = 8 weeks	
			N = 61	Duration = 60 min/session	Mood States, The “multiple sit-to-stand test”, The trunk curl static endurance test
			Female = 61	Frequency = 3 session/week	
			Exp (n = 32)	Intensity = 60%-75%	
			Age (yr) = 49 (7)	Con = Conventional care	
			Con (n = 29)		
			Age (yr) = 47 (8)		
Courneya et al. 2003 [40]	RCT	Early stage Breast cancer	Characteristics of cancer treatment = Chemotherapy – Radiotherapy	Exp = Aerobic exercise	FACT- G, FACT-B, FACT-F
			N = 52	Length = 15 weeks	
			Female = 52	Duration = 35 min/session	
			Exp (n = 24)	Frequency = 3 session/week	
			Age (yr) = 59 (5)	Intensity = 70%-75%	
			Con (n = 28)	Con = Conventional care	
			Age (yr) = 58 (6)		
Ergun et al. 2013 [41]	RCT	Breast cancer (stages I-IIIA)	Characteristics of cancer treatment = Chemotherapy, radiotherapy, mastectomy, axillary dissection and sentinel lymph node biopsy	Exp = Aerobic exercise and resistance training.	EORTC QLQ-C30, BFI, BDI, ELISA kit, RayBio Human
			N = 60	Length = 12 weeks	
			Female = 60	Duration = 45 min/session	Cytokine Antibody Array 3
			Exp (n = 20)	Frequency = 3 session/week	
			Age (yr) = 49.65 (8.25)	Intensity = 60%-80%	
			Home-based exercise (n = 20)	Con = Home-based exercise (brisk walking for 30 min/day for 3 days/week) + education programme	
			Age (yr) = 55.05 (6.85)	Education group = patient information booklet that also included lymphedema-specific exercises	
			Education group (n = 20)		
			Age (yr) = 55.30 (10.37)		
Milne et al. 2008 [42]	RCT	Early stage Breast cancer	Characteristics of cancer treatment = Chemotherapy – Radiotherapy	Exp = Aerobic exercise, resistance training and stretching.	FACT-B, SCFS, rPARQ, Aerobic Power Index
			N = 58	Length = 12 weeks.	
			Female = 58	Duration = 30 min/ses.	
			Exp (n = 29)	Frequency = 3 ses/wk.	
			Age (yr) = 55.2 (8.4)	Intensity = about 75%.	
			Con (n = 29)	Con = Delayed exercise group	
			Age (yr) = 55.1 (8.0)	(DEG) completed the exercise program from 13 to 24 weeks.	
Mutrie et al. 2007 [43]	RCT	Early stage Breast cancer	Characteristics of cancer treatment = Chemotherapy – Radiotherapy and combination	Exp = Aerobic exercise and resistance training.	FACT-G, FACT-B, FACT-F, BDI, PANAS, SPAQ Leisure time, BMI, 12 minute walk test
			N = 174	Length = 12 weeks.	
			Female = 174	Duration = 45 min/ses.	
			Exp (n = 82)	Frequency = 2 ses/wk.	
			Age (yr) = 51.3 (10.3)	Intensity = 50%-75%.	
			Con (n = 92)	Con = Conventional care.	
			Age (yr) = 51.8 (8.7)		
Saarto et al. 2012 [44]	RCT	Early stage Breast cancer	Characteristics of cancer treatment = Chemotherapy – Radiotherapy	Exp = Aerobic exercise	EORTC QLQ-C30, FACIT-F, RBDI, WHQ
			N = 500	Length = 48 weeks.	
			Female = 500	Duration = 60 min/ses.	
			Exp (n = 263)	Frequency = 1 ses/wk.	
			Age (yr) = 52.3 (36–68)	Intensity = 86%-92%.	
			Con (n = 237)	Con = Encourage to maintain their previous level of physical activity and exercise habits.	
			Age (yr) = 52.4 (35–68)		
Segal et al. 2001 [45]	RCT	Early stage Breast cancer	Characteristics of cancer treatment = Chemotherapy	Exp = Aerobic exercise	FACT-G, FACT-B, MOS SF-36
			N = 123	Length = 26 weeks.	
			Female = 123	Duration = No reported.	
			Exp (n = 42)	Frequency = 3 ses/wk.	
			Age (yr) = 51.4 (8.7)	Intensity = 50%-60%.	
			Con (n = 41)	Con = Conventional care.	
			Age (yr) = 50.3 (8.7)	Self-Directed Exercise Group = 5 times per week progressive walking at 50% to 60% maximal predicted oxygen uptake.	
			Self-Directed Exercise		
			Group (n = 40)		
			Age (yr) = 51.0 (8.7)		
Winters et al. 2012 [46]	RCT	Breast cancer (stagesI-IIIA)	Characteristics of cancer treatment = Chemotherapy – Radiotherapy	Exp = Resistance training	SCFS, 1-RM, PPB, Hand grip dynamometry
			N = 106	Length = 1 year.	
			Female = 106	Duration = 60 min/ses.	
			Exp (n = 52)	Frequency = 2 ses/wk.	
			Age (yr) = 62.3 (6.7)	Intensity = 60%-80%.	
			Con (n = 54)	Con = Stretching placebo program.	
			Age (yr) = 62.6 (6.7)		

Beck Depression Inventory, BDI; The Brief Fatigue Inventory, BFI; DXA (Dual-energy X-ray Absorptiometry); European Organization for Research and Treatment of Cancer Quality of Life Questionnaire, EORTC QLQ-C30; Finnish modified version of Beck’s 13-item depression scale, RBDI; Functional Assessment of Cancer Therapy, FACT – Breast (FACT-B), Fatigue (FACT-F), General (FACT-G); Functional Assessment of Chronic Illness Therapy (FACIT) questionnaire for fatigue (FACIT-F); Medical Outcomes Study Short Form, MOS SF-36; Multidimensional Fatigue Inventory, MFSI-SF; Physical Activity Readiness Questionnaire, PARQ; Physical Performance Battery, PPB; Piper Fatigue Scale, PFS; Positive And Negative Affect Scale, PANAS; Scottish Physical Activity Questionnaire, SPAQ; Schwartz Cancer Fatigue Scale, SCFS; Satisfaction with Life Scale, SWLS; Women’s Health Questionnaire, WHQ.

*Age presented with mean and SD or range where reported.

**Supervised physical activity interventions usually consisted of a warm-up period, aerobic training (walking, cycling-ergometers and circuits), muscle strength training (chest and leg curls), stretching exercises and a cool-down and relaxation period.

### Characteristics of supervised exercise interventions

Aerobic training was prescribed in all trials (*n* = 9) [38-46], six of which included resistance training [38,39,41-43,46]. Stretching exercises were performed in one study [42]. Supervised exercise interventions had a mean length of 21.4 weeks (SD 15.8) with a mean duration of 44.3 minutes (SD 15.2) and an average of 2.5 (SD 0.7) sessions per week. Training intensity varied substantially among studies, ranging from 50% to 80% maximal heart rate (Table 2).

### Adverse effects

No major adverse effects were reported among studies. Courneya et al. [40] reported five adverse events in the exercise group (lymphedema, gynecologic complaints and influenza), while two adverse events (foot fracture and bronchitis) occurred in the control group. Cantarero et al. [39] reported discomfort or low-intensity pain/stiffness after an exercise session in 3 patients; however, these patients completed the exercise program. Conversely, Ergun et al. [41] and Winster et al. [46] reported no adverse effects, including lymphedema, with exercise interventions.

### Pooled effects estimates for outcome measures

#### Cancer-related fatigue (CRF)

**Figure 2:**
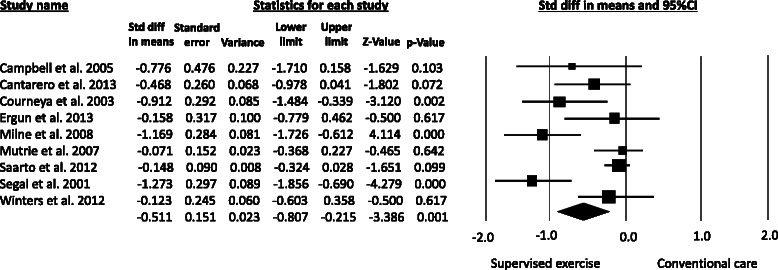
**Metaanalysis for the effect estimate of supervised exercise on CRF in Breast cancer survivors.** Standardized mean difference (SMD) was calculated for the Random effects model of metaanalysis. IV, inverse of variance; CI, confidence interval.

**Figure 3:**
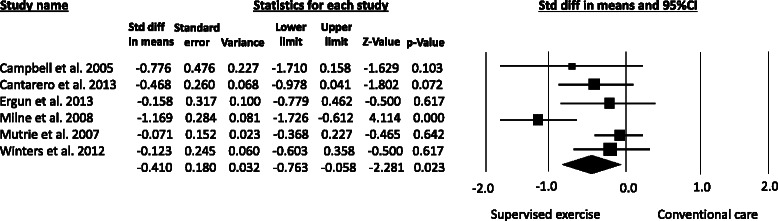
**Metaanalysis for the effect estimate of supervised resistance training on CRF in Breast cancer survivors.** Standardized mean difference was (SMD) calculated for the Random effects model of metaanalysis. IV, inverse of variance; CI, confidence interval.

Pooled analysis demonstrated that supervised aerobic exercise was statistically more effective than conventional care in improving CRF among breast cancer survivors (SMD = −0.51, 95%CI −0.81 to −0.21), with high statistical heterogeneity (*P* = 0.001; I^2^ = 75%) (Figure 2). Regarding subgroup analysis, the pooled SMD for supervised resistance training was −0.41 (95%CI −0.76 to −0.05), indicating a moderate reduction in fatigue from this mode of training (Figure 3). The effect of stretching exercise on CRF levels was addressed by only one study [42], preventing the calculation of pooled effect estimates for this mode of training.

### Meta-regression: heterogeneity and dose–response interaction

**Figure 4:**
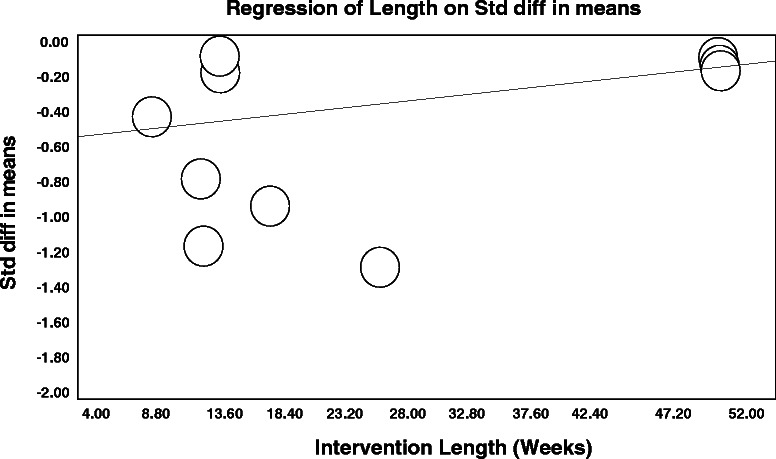
**Bubble plot for the dose–response relationship between the intervention length (weeks) and effect estimates changes for CRF from the nine randomized controlled trials included in the meta-regression analysis (*P*** **= 0.02).**

Our meta-regression analysis showed that publication year (*P* < 0.0001) and the length (*P* = 0.02) (Figure 4), duration (*P* < 0.0001), and frequency (*P* < 0.0001) of the supervised exercise interventions were significantly associated with reductions on fatigue levels. No significant dose–response interaction was observed for training intensity (*P* > 0.05).

### Publication bias

Moderate evidence of publication bias was detected for the effects of supervised exercise interventions on CRF by the Egger’s test (*P* = 0.04).

### Effects of supervised exercise on CRF based on cancer treatment stage

**Figure 5:**
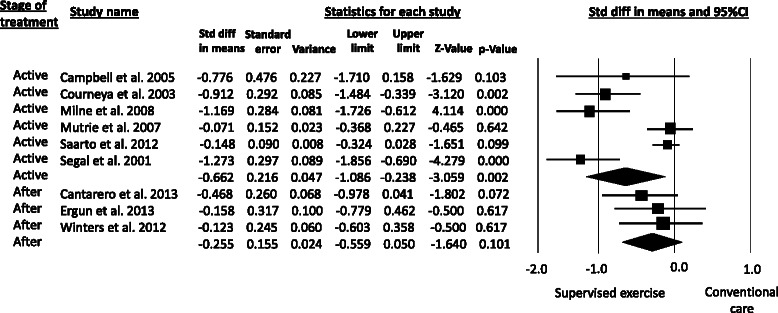
**Metaanalysis for the effect estimate of supervised resistance training on CRF in Breast cancer survivors according to the anti-cancer treatment stage.** Standardized mean difference was (SMD) calculated for the Random effects model of metaanalysis. IV, inverse of variance; CI, confidence interval.

Five studies [38,40,42,43,45] evaluated the effects of supervised exercise on CRF in breast cancer receiving active anti-cancer treatment (i.e., chemotherapy, radiotherapy, hormone therapy or combination). The subgroup analysis showed significant benefits from supervised exercise during active treatment (SMD = −0.66, 95%CI −1.08 to −0.23), high statistical heterogeneity was detected (*P* = 0.002; I^2^ = 83.6%). Four studies implemented supervised exercise in breast cancer survivors after anti-cancer treatment [39,41,44,46]. The pooled effect was not statistically (SMD = −0.25, 95%CI −0.55 to 0.05) with high statistical heterogeneity (*P* = 0.10; I^2^ = 76%) (Figure 5). Time since diagnosis was not consistently reported by authors, although most of the studies recruited women who were beyond five years since primary cancer diagnosis. Hormone therapy included Tamoxifen and aromatase inhibitors. See Table 3 for further details.

**Table 3:** **Effect size estimates for comparisons and secondary outcomes included in the meta-analysis**

Outcome	Effect size random effects model –SMD* (95%CI)	Statistical heterogeneity (I^*2*^)
**Primary outcome (CRF)**		
Supervised aerobic exercise	−0.51, 95%CI [−0.81, −021], (P = 0.001) †	75%
Supervised resistance training	−0.41, 95%CI [−0.76, −0.05], (P = 0.02) †	64%
Supervised exercise during active anti-cancer treatment	−0.66, 95%CI [−1.08, −0.23], (P = 0.002) †	78.6%
Supervised exercise after anti-cancer treatment	−0.25, 95%CI [−0.55, 0.05], (P = 0.10)	85.8%
**Secondary outcomes**		
Depression	−0.23, 95%CI [−0.55, 0.09], (P = 0.16)	69%
Body mass index	−0.14, 95%CI [−0.38, 0.11], (P = 0.28)	0%
Physical activity level	1.10, 95%CI [−0.41, 2.62], (P = 0.15)	85%
**Health-related quality of life**		
Physical wellbeing	0.63, 95%CI [0.08, 1.18], (P = 0.02) †	89%
Functional wellbeing	0.60, 95%CI [0.08, 1.11], (P = 0.02) †	89%
Social wellbeing	0.08, 95%CI [−0.11, 0.27], (P = 0.24)	28%
Emotional wellbeing	0.30, 95%CI [−0.05, 0.65], (P = 0.09)	76%

*Standarized mean difference.

† Significant differences observed (P < 0.05).

Cancer-related fatigue, CRF.

### Results for secondary outcome measures

As shown in Table 3, supervised exercise interventions significantly improved functional and physical wellbeing, but no significant effects were observed for social and emotional well-being domains. There were no significant differences between the supervised exercise group and the control group in depression, BMI and physical activity level (*P* > 0.05).

## Discussion

Our meta-analysis revealed that supervised exercise has a favorable effect on cancer-related fatigue when compared with conventional care and it can be considered as a safe therapy for the management of fatigue and other domains of quality of life in breast cancer survivors. These findings are in accordance with those recently reported by Velthuis et al. [20] and Cramp et al. [17], who found that exercise improved the psychosocial and physical outcomes in cancer survivors during and after treatment. Buffart et al. [47] recently stated that it is necessary to continue studying the guidelines for exercise prescription for cancer patients, specifically regarding the type, localization and side effects related to treatment.

In our subgroup analysis, resistance training significantly improved CRF (SMD = −0.55; 95%CI, −1.09 to −0.01). Similar results have been published in the literature. Milne et al. [42] reported that resistance training produced important benefits on CRF and muscular strength in breast cancer survivors after adjuvant therapy. Similar findings were confirmed by Yuen and Sword in 2007 [48]. In a recent meta-analysis, Strasser et al. [49] found that resistance training during active treatment produced important gains in muscular strength and body composition. Muscular strength was not evaluated in our study due to the large differences in the reports obtained from the studies included. Only one study examined the effects of stretching exercise programs and found it to be beneficial [42].

It has been reported that supervision plays an important role in the benefits of exercise among breast cancer survivors [20]. The mechanism underlying the benefit of supervision could be attributed to improvements in adherence and intensity, perhaps because of greater encouragement or confidence when the help of a health professional is available. In 2009, Whitehead and Lavelle [50] reported that breast cancer survivors preferred supervised exercise training compared to unsupervised exercise. Recently, Markes et al. [51] compared supervised and non-supervised exercise in breast cancer survivors and reported non-significant differences between groups, although the authors reported significant improvements in fitness and daily activities. In light of this, our results demonstrate a favorable tendency in favor of supervised interventions, although our recommendations need to be confirmed by larger randomized controlled trials.

When examining statistical heterogeneity, we found significant positive impacts on CRF with increasing length, duration and frequency of the supervised exercise interventions. Meta-regression analysis showed than exercise interventions performed for more than 28 weeks, nearly 3 sessions per week and lasting 40 minutes per session exert larger effects that low-volume exercise interventions. These dose–response relationships are in agreement with two recent meta-analyses published by Brown et al. [11] and Strasser et al. [14]. On the contrary, we observed no statistically significant dose–response relationship between high intensity (>80% maximal heart rate) of supervised exercise and CRF in breast cancer survivors, even though a strong body of research from previous meta-analyses have demonstrated that high-intensity aerobic and resistance training can provide larger effects than aerobic exercise alone on CRF [11,14,17,20]. Hence, further research is needed to elucidate the role of supervised exercise intensity and the optimal dose of exercise in the management of CRF in women with breast cancer.

An additional relevant finding related to this meta-analysis is that we observed significant benefits on several domains of quality of life (physical and functional well-being) in breast cancer survivors following supervised exercise (see Table 3). These results are consistent with those recently reported by Mishra et al. [52] in a recent Cochrane review concerning exercise and quality of life in cancer survivors. The authors concluded that exercise improves some health-related quality of life domains, such as functional well-being, cancer-specific concerns (e.g., breast cancer), anxiety, fatigue, and other outcomes. Interestingly, the authors encouraged further research to investigate the effects of different training modalities. On the other hand, no evidence of any effect was observed for depression (*P =* 0.16), body mass index (*P =* 0.28) and physical activity level (*P =* 0.15). This lack of significance could be explained by the small number of studies that reported effect estimates for these outcomes and the evident clinical heterogeneity in their measurement. Conversely, other studies have reported consistent changes in depression after exercise interventions in cancer survivors [53].

Our study has several limitations. Emerging evidence has suggested that physical exercise can improve systemic inflammation in cancer survivors [54-57], and it is widely known that cytokines and inflammatory markers are associated with CRF levels [58], though not all studies agree [59-61]. Additionally, it was not possible to evaluate the changes on inflammatory markers following supervised exercise, since only Ergun et al. [41] reported data of the inflammatory markers; therefore, further trials are required to achieve consensus on this topic. The statistical heterogeneity of our results can be attributed to the variability in reporting of several outcome measures (i.e., fatigue, depression, data for quality of life, etc.), intervention procedures and tools used. This reporting heterogeneity and the low availability of data from the studies prevented the analysis of other outcomes, such as muscular strength, and blood biomarkers, including inflammatory cytokines, leptin, glucose-related markers and other tumoral markers. The observed heterogeneity in reporting procedures leads us to recommend that further clinical trials be conducted in a more uniform way in order to achieve strong consensus about the effects of exercise training for breast cancer survivors.

## Conclusions

In summary, our findings demonstrate that supervised exercise could be considered a safe and effective intervention in improving cancer-related fatigue among breast cancer survivors. On the basis of our results, we recommend that supervised and structured exercise programs be prescribed to breast cancer survivors, regardless of treatment stage as a means to improve cancer-related fatigue and some domains of overall quality of life. Further research is required to strengthen this evidence.

## Annexes

### Appendix 1. Search strategy details

1. randomized controlled trial [Publication Type]
2. controlled clinical trial [Publication Type]
3. randomi*ed [Title/Abstract]
4. trial [Title]
5. “clinical trials as topic” [MeSH Major Topic]
6. #1 OR #2 OR #3 OR #4 OR #5
7. Breast cancer [Title/Abstract]
8. (tumour* or tumor*) [Title/Abstract]
9. carcino* [Title/Abstract]
10. #7 OR #8 OR #9
11. Exercise [Title/Abstract]
12. Physical activity [Title/Abstract]
13. Aerobic [Title/Abstract]
14. Resistance [Title/Abstract]
15. Strength [Title/Abstract]
16. Flexibility [Title/Abstract]
17. Stretching [Title/Abstract]
18. #13 OR #14 OR #15 OR #16 OR #17
19. fatigue [Title/Abstract]
20. Cancer-related fatigue [Title/Abstract]
21. #19 OR #20
22. #6 AND #10 AND #18 AND #21
